# Exploring Attitudes to Lung Cancer Screening in England: An Inductive Content Analysis of Online Commentary Following Media Announcement of a National Lung Cancer Screening Programme

**DOI:** 10.1111/hex.70568

**Published:** 2026-03-23

**Authors:** Alice Milne, Jodie Chalmers, Samantha L. Quaife, Anna Bibby

**Affiliations:** ^1^ Academic Respiratory Unit, University of Bristol Bristol UK; ^2^ Centre for Cancer Screening, Prevention and Early Diagnosis, Wolfson Institute of Population Health Queen Mary University of London London UK

**Keywords:** attitudes, barriers, qualitative research, screening, stigmatisation

## Abstract

**Introduction:**

On 26 June 2023, National Health Service (NHS) England announced plans for a national lung cancer screening programme, sparking significant online discussion and debate. Whilst screening has been shown to improve mortality in lung cancer, uptake is lower than desired. We hypothesised that online public responses to national news articles may provide an honest insight into public perceptions of lung cancer screening, particularly among those who may be underrepresented in research. The aim of this study was to explore online attitudes to lung cancer screening, identifying potential barriers and facilitators to screening participation.

**Methods:**

This qualitative content analysis involved a targeted online search of the major UK news outlets, and their affiliate social media sites for articles published between 19 June 2023 and 26 July 2023. Seventeen relevant articles were included, and 921 comments were extracted. Inductive content analysis was used to analyse the data.

**Results:**

A variety of attitudes and perspectives on the announcement of lung cancer screening were identified which were organised into four categories: Stigmatisation of smoking behaviour; Feasibility of NHS delivery; Eligibility and prioritisation; Scepticism and misinformation. Comments were often contextualised by personal health experiences, societal influences, and the politicisation of healthcare.

**Conclusion:**

This study identifies a barrier to lung cancer screening participation that has not been reported in previous work – concern regarding NHS capacity. It builds upon previously identified themes including screening misinformation, and scepticism. The prevalence and strength of smoking‐related stigma, and the associated internalised shame expressed by people who smoke, may help explain the reduced uptake of lung screening seen in this group. The inclusion of voices not previously captured in screening uptake research builds on existing knowledge from prior qualitative work and provides a fuller picture of the personal and societal barriers to lung screening attendance. Interventions aiming to improve informed participation in screening need to consider both individual‐ and societal‐level barriers to engagement.

**Patient or Public Contribution:**

The data incorporated within this study are entirely derived from comments and opinions shared on social media and news outlet comment sections. Comments are anonymous, and hence sources cannot be traced, but commenters are likely to encompass eligible screening participants, relatives, and the general public. By utilising online comments, we aimed to capture voices not previously included in lung cancer screening uptake research, helping to identify novel perspectives on the screening programme.

## 1 Introduction

Lung cancer is the most common cause of cancer mortality, with the majority of patients presenting with advanced‐stage disease, when treatment options are limited [1, 2, 3]. Early diagnosis, i.e. at stage I or II, is associated with higher rates of radical treatment and better prognosis, but a lack of symptoms in the early stages makes this challenging [1]. The US National Lung Screening Trial (NLST) demonstrated that screening high‐risk, asymptomatic individuals with low‐dose computed tomography (LDCT) significantly increased the detection of early‐stage lung cancer and reduced lung cancer mortality [4]. Further studies including the Belgian‐Dutch randomised controlled trial ‘Nederlands–Leuvens Longkanker Screenings Onderzoek’ (NELSON) and the UK Lung Cancer Screening study (UKLS) confirmed these findings, and demonstrated cost‐effectiveness [5, 6, 7]. Consequently, the UK National Screening Committee recommended lung cancer screening [8], and on 26 June 2023, the UK government issued a press release announcing a national targeted lung cancer screening programme [9].

The success of any screening programme is dependent on engagement and uptake within the target population. Uptake of lung cancer screening invitations is consistently around 50%, including in trials specifically designed to maximise participation, such as the Lung Screen Uptake Trial (LSUT), the ‘SUMMIT’ longitudinal cohort study, and the Yorkshire Lung Screening Trial [10, 11, 12]. Real‐world data from NHS England's Targeted Lung Health Check programme is similar, with an uptake rate of 53% reported in September 2024 (unpublished Targeted Lung Health Check National Programme Activity data, courtesy of North of England Care System Support [NECS] consultancy analytics). Unfortunately, the populations at highest risk of non‐participation in screening are also those at the highest risk of developing lung cancer [1, 13, 14]. People who smoke, and people experiencing socioeconomic deprivation, are the least likely to participate in lung cancer screening. Unless this is addressed, poor uptake threatens to undermine lung cancer screening and worsen existing health inequalities [15].

Barriers to screening uptake can be practical and psychological. Psychological barriers include avoidance, fatalism and anxiety [16]. Practical barriers may include a lack of transport, cost, caring responsibilities, work, and other commitments [17]. Efforts have been made to address these obstacles, for example, through varying appointment times, screening within community settings such as supermarket car parks, and subsidising transport costs.

Prior research into lung screening uptake has used participant questionnaires, qualitative interviews and focus groups [17, 18, 19]. In this study, we collected data in the form of anonymous online comments on lung cancer screening news articles, hoping to obtain novel perspectives from voices that may not be represented in more traditional research approaches.

### 1.1 Research Aim

The aim of this study was to explore attitudes to lung cancer screening through analysis of online comments, identifying potential barriers and facilitators to screening participation.

## 2 Materials and Methods

### 2.1 Identifying Sources

We conducted a qualitative content analysis of online comments and responses to national news articles between 19 June 2023 and 26 July 2023, to capture articles published 1 week prior and up to 1 month after the announcement of the national lung cancer screening programme on 26 June 2023. All articles identified that were relevant to the announcement were published between 21 June 2023 and 26 July 2023. The extended window was searched to ensure that no articles were missed. Authors conducted a second check, 2 months later, with no additional papers identified.

### 2.2 Researcher Reflexivity

The experience of the authorship team is varied. AM (BA, MSc) is a qualitative researcher working in a respiratory research unit; SQ (BSc, MSc, PhD, CPsychol) is an experienced behavioural scientist and chartered psychologist; JC (BSc, MB BChir, MRCP) and AB (BSc, MBChB, PhD, MRCP, DTM&H) are respiratory physicians and clinical academics. All authors are female. AB and SQ have a wealth of experience in the sphere of lung cancer research and the implementation of the lung screening programme. The combination of perspectives has helped to strengthen our analysis through the inclusion of nuanced experiences and interpretations.

### 2.3 Data Collection

**Figure 1:**
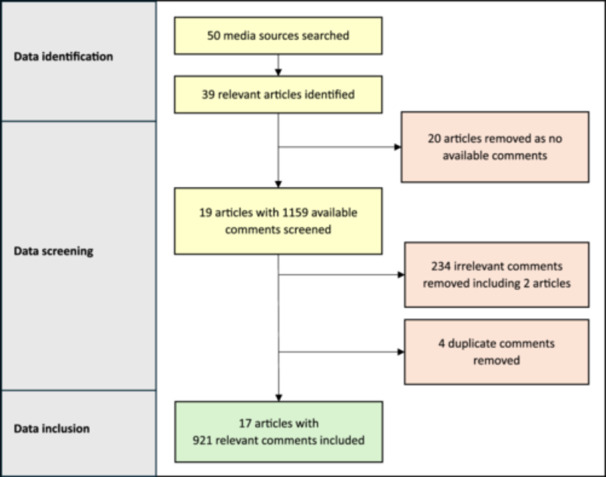
PRISMA flow diagram of data identification, screening and inclusion.

Articles were identified through an online search, using the top three search engines: Google, Bing & Yahoo, with screening‐related terms, including ‘Targeted Lung Health Check’, ‘Lung Cancer Screening’ and ‘National lung screening programme’. In addition, the 41 most accessed online news outlets in the UK, and their affiliate social media sites were searched specifically for eligible articles [20]. We used the advanced search function of social media site ‘X’ with the same search terms to filter for exact dates and phrases. Online news sites and their affiliate ‘X’ social media accounts were considered separate sources for the purposes of data identification; there was no overlap of comments between news sites and their affiliate ‘X’ accounts; this was verified by a text similarity search. When duplicate comments were identified, the first comment was included in the analysis, and the duplicate comment was excluded (Figure 1).

Articles were included if they contained content relevant to the national lung cancer screening announcement, had associated comments, and were publicly accessible using the internet from the United Kingdom. To view the article and associated comments, The Times and The Telegraph required a subscription; The Metro, The Express, The Mail Online, The Mirror and The Nottingham Post required acceptance of personalised advertisements or a subscription; ‘X’ required a login. Comments on news sites and social media were subject to platform policies, including the right to comment removal and account suspension if comments were felt to breach community standards. Comments moderated before the data search would not have been visible. News sites did not consistently confirm whether removed comments would be flagged. ‘This comment has been removed’ was present once in The Telegraph comments section; it was not clear whether removal was by the news site or the commenter. There were no other references to comment removal. Authors made accounts where required, to view the full article and comments; no articles were inaccessible. Replies to comments were visible and were included. Comments were reviewed and those deemed irrelevant to the study' aims (i.e. off‐topic ‘whataboutery’, overt trolling, unrelated political commentary) were discounted (Figure 1).

### 2.4 Consent and Ethical Approval

Consent processes were not applicable for this study as there was no direct participant involvement. Research ethics approval for the study was waived as comments were publicly available and had been voluntarily uploaded to an open forum without expectation for privacy or concealment. Additionally, there was no study‐specific data generation, and researchers had no contact with patients or members of the public in any capacity for this study. There is a precedent of previous studies with a similar methodology, in which research ethics approval was considered unnecessary [21].

### 2.5 Data Analysis

**Figure 2:**
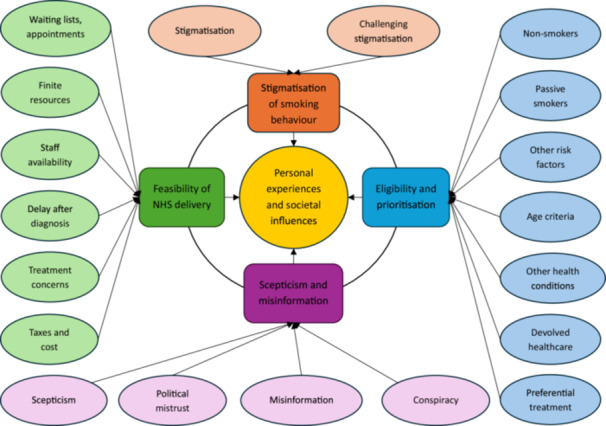
Coding Tree. The four dominant categories: Stigmatisation of smoking behaviour; Eligibility and prioritisation; Scepticism and misinformation; Feasibility of NHS delivery, surrounded by codes, and framed within personal experiences and societal influences.

All eligible comments, including primary comments, sub‐comments and replies, were collated and imported into NVivo 15 qualitative data management software. The characteristics of the associated news articles were gathered and stored electronically to provide contextual data. An inductive content analysis was conducted, structured according to Elo et al.'s three‐phase approach [22]. Categories were derived from the data and were not identified in advance. The phases were as follows:

- Preparation Phase: During this initial phase, the dataset was considered as a whole. Authors familiarised themselves with the comments through data immersion. Notes and codes were not introduced at this stage.
- Organising Phase: AM conducted open coding within NVivo software to develop a descriptive account of the content. Units of meaning were noted throughout the data, and one or multiple codes was added to each; codes were freely added to all data. Separately, JC and AB conducted open coding in NVivo and Microsoft Excel. Code headings were then shared and reviewed by the authors. Disagreements within the research team were resolved by returning to the source data and establishing a majority vote between the authors. In the case of continued disagreement, SQ, as a psychologist and senior social scientist, was elected to make the final decision (although this was not needed). The specific unit of analysis for this study was ‘lung cancer screening attitudes within online comments’, and subsequent category grouping was informed by this. Through interpretation of meaning, subcategories were grouped under larger headings, therefore condensing the data into main categories – termed by Elo et al. as ‘abstraction’. Care was taken to ensure that the final categories constructed were reflective of the breadth of content across the dataset (Figure 2).
- Reporting Phase: Methodological rigour and credibility was considered by authors throughout analysis and reporting. Research ‘trustworthiness’ was achieved through adherence to the trustworthiness checklist for content analysis [23], and rigour was maintained through referring to the COREQ checklist [24]. Whilst our primary objective was to analyse the data qualitatively, we have also reported descriptive statistics and quantitative values to depict distribution of comments for the purposes of transparency. Rigorous adherence to the analytic process throughout, and transparency in reporting provide methodological credibility and legitimacy to results [23, 25, 26]. For the purposes of research integrity and conformability, quotations are provided within the body of the text as source data, demonstrating how authors' analysis and interpretation reflect the data. For transparency in reporting, all quotes are published in their original form to retain data integrity, aside from removal of names to protect anonymity of participants; spelling and grammar have not been amended.

## 3 Results

After final data screening, a total of 921 comments from 17 online news articles were included for data analysis; data sources are listed in Table 1. Data was analysed in line with the study objectives and 4 categories were identified, reflecting the range of opinions and perspectives expressed in the data. The four categories are: Stigmatisation of smoking behaviour; Feasibility of NHS delivery; Eligibility and prioritisation; Scepticism and misinformation.

**Table 1:** Data sources.

News source	Article title	Date	Available comments	Included comments
1. The Telegraph (news site)	NHS lung cancer tests for every‐smoker	21/06/2023	732	588
2. The Telegraph (X)	NHS lung cancer test for every ex‐smoker	21/06/2023	4	3
3. The Mail Online (news site)	NHS plans to offer cancer tests to every ex‐smoker to boost cancer survival rates	22/06/2023	131	92
4. The Express (news site)	Every ex‐smoker to be offered lung cancer tests in new NHS programme	22/06/2023	15	11
5. The Times (news site)	Lung cancer campaign will give scan to anyone who has smoked	22/06/2023	72	66
6. Department for Health & Social Care (X)	Targeted lung cancer screening programme. We're rolling out a targeted lung cancer screening programme across England to help detect cancer earlier and speed up diagnosis.	26/06/2023	26	20
7. The Southend Echo (news site)	Free lung cancer screenings for smokers in England	26/06/2023	3	3
8. The Bournemouth Echo (news site)	Free lung cancer screenings for smokers in England	26/06/2023	1	1
9. Stroud News and Journal (news site)	Free lung cancer screenings for smokers in England	26/06/2023	1	1
10. The Metro (news site)	Rishi Sunak defends NHS cancer services ‘lagging behind other countries’	26/06/2023	3	1
11. The Mirror (news site)	Targeted lung cancer screening roll out could save thousands from silent killer	26/06/2023	2	2
12. Nottinghamshire Live (news site)	Rishi Sunak visits Nottingham to announce extension of lung cancer screening	26/06/2023	10	7
13. Sky News (X)	‘I want to make sure the NHS is fit for future’ Rishi Sunak says a new screening programme for lung cancer will save ‘thousands of people's lives’	26/06/2023	116	87
14. The Times (news site)	NHS lung cancer tests to catch 9,000 cases a year ‐ National screening programme will target people aged between 55 and 74	26/06/2023	17	16
15. The Telegraph (news site)	NHS lung cancer plans will save 9000 lives a year ‐ Scans being offered to ex‐smokers over the age of 55 will be a lifeline, Rishi Sunak says	26/06/2023	20	19
16. The Times (news site)	Steve Barclay: Lung Cancer Programme will catch thousands of cases earlier	26/06/2023	2	2
17. ITV News (X)	Targeted screening to detect lung cancer sooner to be rolled out	26/06/2023	2	2

**Figure 3:**
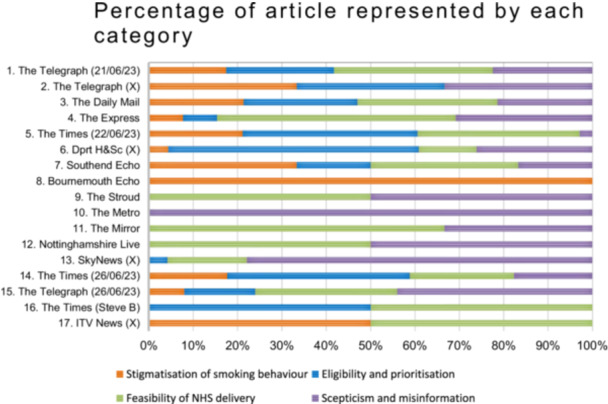
Percentage of article represented by each category.

The breakdown of the number of comments assigned to each category, per article, is depicted in Table 2. The percentage of each article represented by the four categories is depicted in Figure 3. The largest category was Feasibility of NHS delivery, (41.3% of comments), followed by Scepticism and misinformation (33.4% of comments), Eligibility and Prioritisation (29.5% of comments), and finally Stigmatisation of smoking behaviour (19.6% of comments). If comments included remarks pertinent to two categories, they were coded for both, meaning that total percentages exceed 100%.

**Table 2:** Number of comments assigned to each category, per article.

	Stigmatisation of smoking behaviour	Eligibility and prioritisation	Feasibility of NHS delivery	Scepticism and misinformation
1. The Telegraph (21/06/23)	129	180	265	166
2. The Telegraph (X)	1	1	0	1
3. The Daily Mail	25	30	37	25
4. The Express	1	1	7	4
5. The Times (22/06/23)	15	28	26	2
6. Dprt H&Sc (X)	1	13	3	6
7. Southend Echo	2	1	2	1
8. Bournemouth Echo	1	0	0	0
9. The Stroud	0	0	1	1
10. The Metro	0	0	0	2
11. The Mirror	0	0	2	1
12. Nottinghamshire Live	0	0	5	5
13. SkyNews (X)	0	4	17	74
14. The Times (26/06/23)	3	7	4	3
15. The Telegraph (26/06/23)	2	4	8	11
16. The Times (Steve B)	0	1	1	0
17. ITV News (X)	1	0	1	0

News source 1, The Telegraph news site, had significantly more comments than other articles, accounting for 588 or the 921 comments included. Of note, the news sources are ordered in date of publication; the Telegraph published the first news article on the lung cancer screening programme on 21 June 2023.

In addition to the 4 main categories, further subcategories were identified which, after discussion amongst authors, were not considered to fit into distinct higher order categories. They were considered contextual – individuals expressing their opinions on lung cancer screening in the narrative of their personal experiences, and societal expectations.

Screening practicalities were discussed in 44 comments (4.7% of total comments). Participants attempted to clarify logistical details, such as accessing screening, frequency of imaging, and timelines of the screening programme. Similarly, 13 commenters shared their experience of the screening programme.

Can you request them? [1–235]

So when is this going to happen? [5–65]

Agreeing to screening would have invalidated my health/critical illness insurance, and possibly travel insurance, because it is screening carried out on the basis of being a smoker or ex‐smoker. That may apply to others… [1–417]

The concept of choice and autonomy was raised by 16 participants. In the context of smoking, participants explored whether the sale of tobacco products should be banned. There was also discourse surrounding screening attendance and an individual's right to choose; of note, 1 individual challenged this notion, implying a duty to attend screening to avoid a later cancer diagnosis requiring ‘more costly intervention’.

…Let adults decide for themselves after being given advice. [1–313]

You can make the decision to refuse screening, someone else will take your slot. [1–23]

So, I assume, if you subsequently get cancer (god forbid) at a later stage requiring more costly intervention, you will refuse the treatment omn the same grounds? [1–575]

50 participants across seven articles (5.4% of the total comments) expressed support for lung cancer screening. Some individuals reacted positively to the announcement, calling it ‘a great idea’, whilst others expressed intent to attend. Several commenters acknowledged the value of preventative public health measures, for both personal health benefit and system‐level cost benefits. Of note, comments that expressed a positive opinion often also included a remark pertinent to the four main categories, and thus, such comments were assigned a second code.

Great idea. As an ex smoker I would take this offer up. [5–55]

Yes this is brilliant news, but what about the hundreds of thousands who are currently undergoing very poor services for their current cancers and all of those people waiting for operations for years. [1–9]

Good. Proactive care is always cheaper than reactive care. Let's just hope the data doesn't find itself in the hands of life insurance companies. Generally in favour. [1–435]

### 3.1 Stigmatisation of Smoking Behaviour

The words ‘smoking’, ‘smoker’ and ‘smoke’ were directly mentioned in 291 comments (31.6% of total comments), often in a negative manner. Over 100 participants demonstrated smoking stigmatisation, with numerous commenters using derogatory adjectives, such as ‘lazy’, ‘stupid’, and ‘selfish’. A strong sense of blame was evident; individuals were perceived to be wilfully continuing to smoke despite knowing the potential health ramifications. Hence, subsequent lung cancer diagnoses were considered to be ‘self‐inflicted’ and therefore less deserving of NHS treatment and resources. A sense of otherness was created by some comments through repeated, indirect references to people who smoke (‘they’, ‘these people’).

If you stupid enough to smoke in the first place why should they get special screening. [3–29]

Any prospect of ensuring smokers contribute to the cost of the treatment for their self inflicted and selfish habit? [5–16]

The notion of deservedness was reinforced by a belief that people who smoke were actively disadvantaging non‐smokers, by taking up limited NHS capacity. As a result, those who do not smoke felt that they were being ‘punished’.

…What do people who look after themselves get? They are shunted to the back of the queue as always. It doesn't pay to be healthy, work or be a law abiding citizen in this country cos you are punished for being one. [3–36]

So just keep rewarding poor behaviours? which drives up risks, which creates a cycle of unhealthy people. How about reward those that look after themselves, maybe more would. Just a thought. Better than a race to the bottom in my opinion. [1–596]

Relatedly, several negative comments focused on the impact of smoking on others. This took the form of health concerns following second‐hand smoke exposure, as well as the implications of tobacco exposure on the families of people who smoke. The blame for these secondary smoking‐related harms was placed firmly on people who smoke, and used as further evidence for the ‘unfairness’ of a screening programme that focussed solely on the people perceived to be causing the problem.

What about those of us who have had to breathe in the secondary smoke of these idiots!. [2–1]

Why is it confined to those who knowingly ingested toxic chemicals? I spent all my youth being smoked over by first both parents, then my chain‐smoking father. There was no choice involved, but people have been dying of second‐hand smoke cancer who have never smoked. [1–263]

What about passive smokers who were never stupid enough to smoke but had no choice but to breathe. It's not just smokers who get lung cancer. [1–626]

Several comments from self‐identified people who smoke also expressed negative views towards tobacco use, suggesting societal stigma had been internalised. In some cases, this was expressed as self‐blame, or a lack of deservedness of medical care. Some people explicitly stated that they would not attend lung screening as it would waste ‘precious resources’, and if they developed lung cancer it was due to their own ‘stupidness’.

I will not take valuable resources away from someone more deserving. I knew full well what smoking did 10 year ago but still chose to do it. [1–409]

I used to smoke. Well that was my own stupid decision. Do I want to be checked for every possible issue that might arise – no. Do I want to live to such an age I'm just a burden on others – no. Do I want this government and the NHS to continue being frivolous with my money – no. Leave us to live for however long and say thank you for the great time on planet earth. [1–22]

I smoked for over 40 years and think this is a stupid waste of money. [3–32]

Most articles commented on the cost of running a lung health check programme; consequently, discourse about taxes, cost and financial accountability was mentioned in 157 comments, across 10 articles (17.0% of total comments). Some commenters felt that people who smoke should be required to contribute at an individual level, whilst others suggested that tobacco companies should take responsibility. In contrast, several individuals commented on the increased tax paid in the form of tobacco duty by people who smoke, suggesting that they had made greater contributions to NHS funding as a result.

Why the hell is my hard earned been wasted on wasters? Their poor life choices should not be rectified using my money. [1–88]

I'd like to see tobacco companies contributing massively to this, seeing as they peddled a highly addictive cancer‐causing substance decades after knowing its harms. [5–20]

Smokers continue to cost the rest of us a small fortune. [8–1]

…Please don't forget the amount of tax that anybody who has ever smoked has paid on every single pack of cigarettes they buy. [1–15]

Seventy‐nine comments (8.6% of total comments) challenged stigmatisation of smoking behaviour. Individuals commented on misleading advertisement of smoking benefits in the earlier 20th century, and the addictive nature of smoking. Others argued that self‐inflicted conditions extended beyond smoking, with examples including obesity, diabetes, and sports accidents.

There was a time when the government encouraged people to smoke as being good for the nerves ‐ during WW2 for example… [1–270]

Maybe they can scan for judgmentalism too. I quit 15 years ago, I was a teenager and children often make stupid, selfish decisions for a massive array of reasons. [5–21]

And I guess we shouldn't treat car drivers or DIY fans or people who play sport or climb trees or eat too much or do anything at all – what a childish argument. A civilised society cares for its sick. [1–18]

### 3.2 Eligibility and Prioritisation

The eligibility criteria of the proposed lung cancer screening programme were discussed in 272 comments across 11 articles (29.5% of total comments). Commenters questioned whether the screening eligibility criteria were broad enough, and often expressed a sense that they were being unfairly excluded from screening. The phrase ‘what about’ was frequently used by commenters, usually followed by a suggestion for alternative screening eligibility, for example prioritisation of an alternative population or reallocation of resources away from lung cancer screening completely. Concern was expressed by 55 commenters regarding the health risks from second‐hand smoke exposure, often from people who grew up in households with people who smoked and or were exposed in public spaces, e.g. in pubs prior to the UK's 2007 indoor smoking ban.

Just for ex smokers how is that right. What about us that never smoked but had to sit around people who smoked like a chimney. Testing for everyone. [3–63]

Why just smokers? As if they are the only people who's health is needlessly put at risk by this dangerous, filthy habit!… [1–359]

So we have screening for breast and cervical cancer and now for lung cancer, the latter most frequently caused by poor lifestyle, but no screening for the biggest cancer kill of men, prostate cancer?????????????? [15–9]

Exposure to other lung cancer risk factors, including ‘environmental pollution’, asbestos and radon gas, were mentioned in 15 comments as potential additional eligibility criteria. Relatedly, there was an awareness among many commenters that non‐smokers can ‘get lung cancer too’ with 35 participants suggesting that they should be eligible for screening as well. Concerns were also raised by 61 participants regarding both the upper and lower age limits for screening, with 1 individual noting it ‘should be at least 10 years earlier’, and others asking if those over 74 years old were to be ‘ignored’. 5 commenters discussed that the screening programme was ‘restricted’ to England due to devolved healthcare.

And what about those who grew up or live in heavily polluted cities. [1–503]

…The government may as well say ‘we don't care about anybody after 74’. [1–279]

England only again… [5–41]

Prioritisation was a recurrent topic, with multiple comments mentioning a ‘queue’ for healthcare access. There was a belief that lung health screening was utilising, and potentially exhausting, finite NHS capacity. Individuals questioned the fairness of allocating limited resources to those who smoke, suggesting that screening was allowing people who smoke to ‘skip the queue’, lengthening waiting times for others. Commenters also debated the decision to target areas of socioeconomic disadvantage for screening, with 1 person remarking that cancer is equally ‘serious’ for individuals from more affluent backgrounds. Some felt that the entire programme was inappropriate at a time when NHS capacity was limited elsewhere.

Yes Mr/Mrs Smoker, you can jump the queue… or certainly stay ahead of anyone later diagnosed with cancer through no fault of their own. [1‐537]

They chose to ignore health warnings. Treatment should be prioritised for people who have at least tried to look after themselves or have illnesses or conditions that they could have done nothing to prevent. [3–33]

Regarding the economics of the screening programme, commenters highlighted that smoking increases the risk of numerous health conditions. They disputed the fiscal benefit of scanning people who smoke, given the increased risk of other diseases that may require treatment or be life‐limiting. Some also noted that there would be an implication on the ‘welfare state’ if individuals lived longer into retirement age.

I cannot help but wonder what percentage of smokers have treatment but don't give up smoking and wonder if earlier diagnosis would then significantly increase their life expectancy. [5–67]

If they live longer they will cost a lot more in pensions etc. Then probably need other NHS treatments after. [5–11]

Average age of diagnosis with lung cancer is 70. It's a good thing for patients but it won't save money for the taxpayer. [5‐43]

### 3.3 Feasibility of NHS Delivery

Significant doubts were expressed over the capacity of the NHS to deliver a national screening programme; 380 participants across 14 articles discussed costs, resources, staffing and waiting lists (41.3% of total comments). Fatalistic attitudes were expressed towards access to investigations, treatment options, and waiting times for diagnosed cancers. In this context, the idea of undergoing screening was presented as ‘pointless’ as subsequent waiting times would negate any early diagnostic benefit.

What's the point? If they find cancer they'll just leave you to die. [3–2]

Even if they finally manage to scan people the waiting list for treatment will be so long as to render early detection to be pointless as the patients will probably be dead before they get treatment. [1–507]

The anticipation of long waiting lists if lung cancer was detected caused 17 participants to remark on the emotional turmoil associated with receiving a cancer diagnosis including *‘*fear’ and ‘distress’. There was recognition of one of the potential harms associated with screening, that of ‘worry’ and ‘stress’ even if results were ultimately reassuring. Several individuals stated that they would ‘rather not know’ that they had cancer, if treatment pathways could not be accessed in a timely fashion. In some cases, these concerns overlapped with nihilistic views around lung cancer treatment options in general.

Even if there was universal screening and a problem was detected you'd get ill by worry whilst you wait three years for a consultation. Sometimes the less you know the better you sleep. [3–15]

What is the point of diagnosing a disease in thousands of people, thereby causing untold anxiety, if those people are then left to suffer for many months waiting for third rate treatment by the NHS? [1–276]

Views were framed within the widely acknowledged pressures facing the NHS in the post‐pandemic landscape and heightened by concern over capacity due to healthcare worker industrial action occurring at the time. Frustration at lack of access to GP appointments and reference to extensive healthcare waiting lists were mentioned by 68 participants. Some comments provided first‐hand experience of NHS screening delays, confirming concerns voiced by others.

You're joking? The NHS can barely keep up with appointments for obvious health issues. Identified cases will just be left languishing at the end of a waiting list while their life falls apart… [1–500]

…Another initiative' that will get nowhere because of too few resources, money and specialist staff. The NHS is failing in just about every area (…) so how can it effectively add another major screening and treatment programme to its workload? [1–256]

Ok so he's talking about the new screening programme. My husband and myself received our letters telling us the date and time someone would ring us to answer some questions. Still waiting for phone call, 5 weeks down the line… [13‐13]

### 3.4 Scepticism and Misinformation

Scepticism and misinformation was a common theme, noted in 308 comments, across 14 articles (33.4% of total comments). Some participants raised legitimate concerns around the potential harms of screening, with insight into the associated risks. Several commenters shared personal experiences of incidental findings, such as coronary artery disease and pleural plaques. 1 commenter mentioned ‘overdiagnosis’ (diagnosing cancers that would not have caused any health issues had they not been identified) and ‘false positives’ (diagnosing abnormalities that turn out not to be cancer) – acknowledged limitations of all screening programmes.

I feel this could be a total waste of taxpayers money and raise real worries about non existent medical conditions. I was offered a free scan down here in Cornwall. A few days after the scan, they sent me a letter, stating that my lungs were clear, but I had, unknown to me, coronary disease. [5‐5]

And, of course, no unhelpful dwelling on the perils of overdiagnosis, false positives (and negatives) etc. [13–43]

At times, scepticism reflected misunderstandings around screening processes, for example regarding imaging modality (x‐ray *vs.* computed tomography) and scan frequency. Some individuals conflated long GP waiting times with reduced access to screening, reflecting a misperception that individuals had to see their GP to participate.

The idea X ray screening can prevent 1 in 4 lung cancer deaths is simply incredible. Show us the evidence!!! [15‐15]

Unfortunately to be in the queue for a scan you first have to attempt to get a GP appointment which is nearly impossible… [10–1]

Frustration and mistrust in the healthcare system echoed through the data. Screening was viewed by some as a paternalist intrusion, subjecting people to testing that they would not otherwise choose to have. Several individuals did not believe that early diagnosis resulted in improved treatment options for lung cancer; they often exhibited fatalistic perspectives of both screening and lung cancer treatment options.

If people suspect they have an illness they seek help, don't need the govt to constantly interfere in their lives. [3–75]

And provide the same prehistoric treatment, no thanks. [3–65]

The articles that named or included pictures of politicians often attracted angry or mistrustful comments towards political figures and the government. 131 participants across 11 articles expressed political mistrust. The lung cancer screening announcement was dismissed by some commenters as another *‘*soundbite’, a ‘tick‐box exercise’ and a ‘headline’ for political gain. 11 comments also referred to ‘13 years’ ─ the duration that the conservative party had been in government at the time.

Your party took the NHS from being the best in the world to it crumbling before our eyes. Stop gaslighting. No one believes you. [13–46]

The Tories spend 13 years trashing the NHS and then try to position themselves as the best party to fix their damage. The sad thing is some people will fall for it. [13–59]

There was concern about whether lung cancer screening would lead to further privatisation of the NHS, with commenters querying if screening would be facilitated or delivered by private companies. Other responders were more cynical and questioned whether lung cancer screening was being introduced to make the NHS appear more appealing to expedite it being *‘*sold off’ *t*o private companies.

You naive enough to believe that the NHS will be running this? This will be a tory funding private corporation with a markup that would make Louis Vitton blush. [1–352]

And which of his Private Health ‘care’ buddies have got this contract? Have to find more parking for these private screening trucks. [13–36]

As is often observed in online forums, 30 commenters discussed conspiracy theories, particularly around vaccine misinformation: ‘jab‐induced turbo‐cancer’ and ‘DNA harvesting’. Supporting evidence for these comments was not provided, and the tone was often hyperbolic.

This seems like the way to start pushing the cancer ‘vaccines’ that are being developed. Remember, testing is the first stage. Create the fear and the need and then start the ‘safe and effective’ prevention and cure. [1–302]

They don't care about your health, all these tests are just for DNA harvesting, look up Genomic Sequencing and how the UK wants to know that exact data on everyone in the UK. Still Normies will think that they are trying to protect their health, 10 jabs in! [3–81]

## 4 Discussion

This inductive analysis of online responses to the national lung cancer screening programme announcement highlights significant population‐ and individual‐level barriers to screening participation. We identified four categories in the data: stigmatisation of smoking behaviour; eligibility and prioritisation; feasibility of NHS delivery; scepticism and misinformation. There was significant thematic overlap within comments, and many were framed in the context of firsthand experiences, as well as the wider social and political landscape. In this discussion, our findings are interpreted, and compared with existing literature. The following topics will be discussed: Stigmatisation; Deservedness; NHS capacity concerns; Online commentary and media reporting; Practicalities and acceptability of screening; Strengths and limitations; Implications for future research.

### 4.1 Stigmatisation

One of the main categories identified in the data was smoking stigma. Stigmatisation, blame and judgement are recognised barriers to screening attendance and healthcare‐seeking behaviour [27, 28, 29]. Nonetheless, the significant smoking‐related stigma expressed by many commenters, and the strength of feeling associated with it, was striking. Despite its addictive nature, smoking was perceived to be a lifestyle choice and tobacco‐related disease was regarded as ‘self‐inflicted’ [30]. The prevalence of these views and the vehemence with which they were expressed in public forums are likely to result in internalised stigma among current tobacco users. This correlates with the high burden of shame and guilt expressed by people diagnosed with smoking‐related conditions [31], and may be a major contributing factor in the lower lung cancer screening uptake rates seen in people who smoke [17, 19, 32, 33].

### 4.2 Deservedness

The opportunity costs associated with funding a lung cancer screening programme in preference to other healthcare services were recognised, and were a source of frustration. The concept of fairness and ‘deservedness’ arose frequently; many commenters expressed their belief that people who smoke were not deserving of screening, blaming them for choosing to smoke and thereby responsible for any ensuing smoking‐related diseases. Interestingly, phrasing such as ‘reward’, ‘deserve’ and ‘special’, alongside the expressed feelings of injustice around eligibility, implied that lung cancer screening was considered a positive intervention by many. Concern about cost and deservedness of screening for self‐inflicted conditions has been noted as a barrier to lung cancer screening attendance in previous qualitative studies [34, 35]. Healthcare waiting times were referenced repeatedly, and smoking‐related stigma surfaced here too, with resentment expressed towards the perceived preferential treatment being given to people who smoke.

### 4.3 NHS Capacity Concerns

The topic of NHS capacity permeated each of the categories identified. Comments focused on whether lung cancer screening was an appropriate use of resources. In some instances, concerns reflected misconceptions about screening pathways and processes. For example, some people commented that access to screening was contingent on the availability of GP appointments; whilst this may reflect misunderstandings about appointment booking, it is also likely that individuals were expressing frustration about healthcare accessibility more generally. Throughout the categories, misunderstanding about the lung cancer screening programme was common. In an American qualitative study examining non‐participation in lung health screening, misunderstanding of the screening process resulted in some eligible participants opting‐out [36], suggesting significant implications for healthcare miscommunication.

Fatalistic beliefs also related to the NHS' capacity to deliver treatment, rather than the availability of specific cancer therapies. Individuals were concerned that those diagnosed would die waiting for treatment; this can be contextualised by the concurrent sociopolitical climate, with record waiting lists and ongoing industrial action by healthcare workers. Substantial political upheaval occurred in the UK in 2022–2023: the UK prime minister was replaced, and cutting NHS waiting lists was announced as a major government priority [37], likely adding to the public perception of strain on the NHS. Additionally, nurses, resident (formerly ‘junior’) doctors, and ambulance service workers all undertook industrial action between 2022 and 2023 in disputes over pay awards, further increasing waiting lists [38, 39] [40]. Awareness of the pressures faced by the NHS may have impacted participants' willingness to attend screening due to both concern about receiving timely treatment for themselves, and a broader sense of not adding to NHS demand. As a result, it is likely that participants expressed greater concerns about waiting lists and political mistrust than they would have had if sampled in a different sociopolitical climate.

### 4.4 Online Commentary and Media Reporting

Studies have shown that individuals post more disinhibited and aggressive opinions online when they do so anonymously [41]. Comments cannot be traced back to individuals and there are fewer ‘real‐life’ consequences associated with controversial posts. Additionally, website algorithms amplify posts that provoke more engagement, which may encourage posters to exaggerate or overstate their views [42]. It is recognised that people post comments similar in tone to those that have preceded it, matching the ‘social norm’ of the comment thread [41]. Online discourse is often more extreme and opinions more polarised than in face‐to‐face conversation, and our findings should be interpreted in this context [43]. For people deciding whether to attend lung cancer screening, the presence of judgemental comments online may influence decision‐making, even if the stated views do not accurately reflect the posters' true beliefs.

Perhaps unsurprisingly, in this analysis of online comments, negative perspectives were shared more frequently than positive ones. Fatalism was expressed regarding lung cancer and the available treatment options. This presents an important barrier to screening attendance and has been shown to disproportionately affect people who smoke. Current tobacco users are more likely to see lung cancer as an untreatable disease and less likely to believe that early detection results in a better chance of survival [18, 19]. Relatedly, Ali et al. found that people who smoke who chose not to participate in the UKLS trial were more likely to report emotional barriers such as avoidance, fear and anxiety than people who no longer smoke [17]. Interventions to address negative beliefs about lung cancer and to increase awareness of radical treatment options may help reduce non‐participation in this highest risk group [19, 27]. Similarly, Quaife et al. noted reduced uptake in the SUMMIT study (an early lung cancer screening trial), in those with negative perceptions of screening [16]. In a recent survey of 27 healthcare professionals involved with lung cancer screening delivery, ‘not wanting to know’ was recognised as a major cause of screening non‐participation [44].

News outlets have established ideological positions and political leanings, through which content is framed. As a result, neutrality in reporting of public health interventions cannot be assumed. Health attitudes are influenced by biases in news outlet content and may have influenced the nature of comments shared. The articles included in this analysis differed in content, tone and reporting style. Notably, the main headlines of the articles varied significantly, despite conveying the same subject matter. The NHS was included in the headline of 8 of 17 articles analysed, smoking (including derivatives) was referenced in 10 headlines, and politicians or government ministers were directly mentioned in 6 headlines. Headline content may have contributed to the frequency with which certain issues were discussed by commenters. There were also tonal differences in the main body of the articles, including information sources and supporting images. Political and governmental figures were referenced in most articles, while mention of the recommendations of the UK National Screening Committee (UKNSC) was less frequent.

### 4.5 Practicalities and Acceptability of Screening

Participants often raised practical queries regarding the timeline for the screening programme, how it could be accessed, and implications of screening on health insurance. The inquiring nature of such comments may indicate that participants were envisaging themselves or those around them participating in the lung cancer screening, suggesting a willingness to accept the screening programme. Many more individuals discussed who would and would not be eligible. Similar findings regarding eligibility criteria concerns have been reported in previous work: when Bell‐Williams et al. interviewed individuals potentially eligible for lung cancer screening and their relatives, they frequently noted that relatives were concerned with eligibility restrictions regarding age and passive smoking [45].

Comments discussing practicalities imply that participants sought a greater understanding of the screening programme. The concept of acceptability provides a useful lens through which to examine this. The Theoretical Framework of Acceptability of healthcare interventions (TFA) [46] incorporates the concept of ‘intervention coherence’ ─ the extent to which the participant understands the intervention and how it works. It demonstrates the importance of clear communication in aiding understanding and increasing acceptability. Public communication and evidence‐based explanations alongside screening announcements would ensure that eligible individuals are able to make informed decisions about screening participation.

### 4.6 Strengths and Limitations

By analysing anonymous comments from public forums, we have included voices less likely to be heard through traditional research approaches, allowing us to identify important barriers to lung cancer screening participation. The comments included in this study were posted anonymously or pseudonymously. As a result, participant demographics and diversity characteristics are not known, and it is not possible to determine which comments were made by those eligible for lung cancer screening. It is plausible to assume that comments stigmatising smoking were predominantly made by people who do not smoke, and therefore not the target population, but their widely expressed beliefs may be read by screening‐eligible people, affecting their likelihood of participation.

It is important to note that the results found from analysis of online comments are not representative of the wider UK population: those without digital access, without English language literacy, and those unable or unwilling to sign up to accounts or view adverts, would not be able to participate in this study. Additionally, self‐selection bias was evident: participants were those who voluntarily posted an online comment; views may differ from those who chose not to comment.

News source 1 from The Telegraph accounted for 64% of total comments, meaning that it is over‐represented in the analysis compared to the other news sources. Given that it was the first publication to report on the new lung cancer screening programme, it is logical that it would generate more interaction and comments than subsequent articles. Similarly, news source 3, The Mail Online, was the second outlet to publish an article and had the second highest number of comments. There is however an inherent risk of bias when one source contributes much of the data – as discussed above, different outlets may attract different demographics of readers, and may portray content with varying neutrality. Of note, each of the categories identified in the analysis were represented by over 120 comments from news source 1, which strengthens our ability to capture a range of attitudes within a single data source.

### 4.7 Implications for Future Research

When considering future approaches to explore lung cancer screening attitudes, the TFA provides a temporal structure to examine acceptability [46]. Anticipated acceptability explores attitudes prior to personal participation, whilst retrospective acceptability pertains to reflections following participation. Incorporating both facets offers a framework by which healthcare professionals and researchers may explore barriers to acceptability throughout the screening process; this is particularly relevant for lung cancer screening, where participants are invited to re‐attend every 2 years.

## 5 Conclusion

Our findings demonstrate the significant, pervasive stigma related to smoking and highlight its potential to deter people who smoke from participating in screening. Whilst there was awareness of the positive aspects of screening, and appropriate recognition of downsides, misunderstandings about screening processes and lung cancer treatment options were prevalent. When introducing novel public health interventions, such as screening programmes, clear and evidence‐based explanations are fundamental to facilitate informed decision‐making in eligible individuals. Wider societal barriers, including concern about NHS capacity, contribute to negative perceptions of screening. Targeted communication highlighting the economic benefits of screening and improved outcomes from early lung cancer diagnosis provides an opportunity to shift negative attitudes.

## Ethics Statement

This research did not require IRB approval because the analysis is of anonymous, publicly available existing data.

## Consent

Informed consent was not required for this article as no patient information is included. All data are sourced from publicly accessible news outlet comment sections.

## Conflicts of Interest

The authors declare no conflicts of interest.

## Data Availability

The data that support the findings of this study are available in the supporting material of this article.
